# Effect of Humic Acid on Morphology, Fluorescence, and Nutrient Uptake of Spring‐Sown Potato Crop Under Saline Sandy Loam Soil

**DOI:** 10.1155/sci5/6184394

**Published:** 2026-03-01

**Authors:** Muhammad Wasim Haider, Syed Mohsin Abbas, Tanveer Hussain, Muhammad Tahir Akram, Muhammad Waseem, Muhammad Asad Saleem, Alina-Stefania Stanciu, Muhammad Nafees, Crossby Osei Tutu

**Affiliations:** ^1^ Department of Horticultural Sciences, The Islamia University of Bahawalpur, Bahawalpur, 63100, Pakistan, iub.edu.pk; ^2^ Department of Horticulture, Faculty of Agricultural Sciences, University of the Punjab, Lahore, 54590, Pakistan, pu.edu.pk; ^3^ Department of Horticulture, PMAS-Arid Agriculture University, Rawalpindi, 46300, Pakistan, uaar.edu.pk; ^4^ Department of Food Science and Technology, Faculty of Agriculture and Environment, The Islamia University of Bahawalpur, Bahawalpur, 63100, Pakistan, iub.edu.pk; ^5^ Horticultural Research Station, Sahiwal, 57000, Pakistan; ^6^ Department of Agriculture-Horticulture, Faculty of Environmental Protection, University of Oradea, Oradea, 3700, Romania, uoradea.ro; ^7^ Department of Family and Consumer Sciences, University of Ghana, Accra, Legon, Ghana, ug.edu.gh

**Keywords:** biostimulants, crop growth, fluorescence-related metrics, high irradiance, nutrient uptake efficiency, organic soil amendment, salinity, sustainable yield

## Abstract

Soil salinity is one of the most important abiotic stresses that significantly limits agricultural productivity, particularly in hot arid and semiarid areas. In such areas, crops are also subjected to high irradiance, which may exacerbate the physiological stress. The conventional chemical and cultural practices have proved ineffective in achieving the sustainable production of crops under these combined stress conditions. Humic acid has been reported to enhance tolerance of plants to salinity; most of the studies emphasize the generalized physiological responses but with the least information on photosynthetic and nutrient uptake efficiency of potato plants under field conditions characterized by both salinity and high irradiance. The present two‐year investigation aimed to assess the effects of varying humic acid application rates on the growth, fluorescence, yield, and nutrient uptake of potato cv. Santana. The trial was carried out using a randomised complete block design (RCBD) with a factorial arrangement of treatments. Humic acid was soil‐dressed at three rates (1000, 1500, and 2000 kg ha^−1^) and assessed at two different periods (65 and 85 days after sowing), with an untreated control for comparison. The significant (*p* ≤ 0.01) improvements in plant growth, fluorescence, yield, and nutrient uptake were observed with increasing humic acid application rates. However, the highest increases in plant height (89%), number of stems plant^−1^ (95%), number of branches plant^−1^ (49%), number of leaves plant^−1^ (75%), leaf area index (220%), quantum yield of photosystem II (Φ_II_; 130%), chlorophyll content (65%), number of tubers plant^−1^ (115%), average tuber weight (34%), total tuber yield (60%), marketable yield (47%), plant N uptake (36.7%), plant P uptake (73%), N uptake efficiency (50.5%), P uptake efficiency (182% times), and decreased nonphotochemical quenching (Φ_NPQ_; 75%) and nonregulatory energy dissipation (Φ_NO_; 39%) were achieved with the highest application rates of humic acid (2000 kg·ha^−1^) compared to the control. In conclusion, the use of humic acid at 2000 kg·ha^−1^ substantially improved potato growth, yield, photosynthetic efficiency, and nutrient uptake, proving it a promising strategy for sustainable cultivation.

## 1. Introduction

Potato is the third most consumed and the fourth most produced food crop all over the world [1]. It is a rich source of energy due to its high starch and mineral content [2–5]. The autumn crop (Oct–Feb) is the main crop of potato in subtropical regions [3, 6]. However, market prices are low during its harvesting, or the fresh potatoes have high prices later in the period after the end of March, which can be attained through growing the spring crop (Jan–May) [7]. The spring crop is constrained by the rising temperature during March–May (an increase of 2.7°C in the last decade) [8]. The high levels of soil salinity further exacerbate the stress on potato crops [9], especially at the critical tuber initiation stage [10, 11]. Furthermore, several other environmental and agronomic challenges, particularly the overuse of chemical fertilizers and the poor organic content in soils (< 1%), lead to nutrient leaching and reduced soil fertility [3, 12, 13]. These environmental stresses reduce productivity, and conventional practices have failed to sufficiently mitigate these effects. The extreme use of chemical fertilizers to increase tuber yields for more returns is unkind to the earth’s resources [9]. Hence, nutrient management in potato crop depends on less nutrients leaching from potato fields while maintaining tuber yield and quality [14]. While organic farming has deep historical roots, recent trends in agriculture are now focused on optimizing fertilizer use and shifting back towards organic farming practices. This shift is driven by a growing concern for producing healthier, more sustainable food products, reducing reliance on inorganic fertilizers and addressing environmental challenges associated with chemical inputs [15–20]. Likewise, organic farming of potatoes for human use improves human health and nutritional safety [21–25]. Therefore, the establishment of a sustainable, productive, and vibrant organic subsector in potato‐growing countries is largely dependent on effective nutrient management via mineralization of organic matter [26–29].

To date, a wide range of strategies has been implemented to mitigate the adverse effects of environmental stresses, including the use of heat‐ or salt‐tolerant genotypes [30, 31], improvements in irrigation management [32, 33], refined cultivation techniques [34, 35], and the application of biostimulants [36, 37]. Nevertheless, one approach to minimizing the detrimental effects of high temperature and salt stress is the use of organic compounds, e.g., humic acid. Sustainable agriculture involves applying not only the mineral fertilizers that encompass macro‐ or microelements but also biostimulants, which are high in biologically active substances that activate mechanisms to improve nutrient uptake, nutrient efficiency, and tuber quality when applied to the rhizosphere or plants [25, 38]. This definition also includes various organic and inorganic materials, like humic acid, seaweed extract, protein hydrolysates, chitosan, and phosphates.

Humic substances, including humic acid and fulvic acid, refer to the natural compounds that are biologically active in the soil and can stimulate plants through different mechanisms [39]. Humic acid not only influences soil physicochemical parameters and soil microbial biomass, resulting in higher availability of nutrients and organic matter [40–43]. Humic acid is also a naturally occurring compound in the soil and a bioproduct of the decomposition of organic materials, which have been successfully employed in the cultivation of different crops. In field experiments, the direct impact of humic acid on plant growth has been described sufficiently; these impacts include improved nutrient uptake and root growth [44]. The application of humic acid has been illustrated in the cultivation of several crops, including tomato [45], blueberry [46], Hungarian vetch [47], maize [48], and potato [25, 49]. The biostimulant application mitigates the effects of the abiotic stresses in plants by controlling several processes [50]. It improves nutrient uptake under stress conditions [51]. It has also been involved in protecting plants against salt and heat stresses through the synthesis of heat‐shock proteins [52]. Photosystem II (PSII) is generally more susceptible to environmental stresses, particularly heat and high irradiance, than PSI (PSI), and its function can be rapidly impaired under adverse conditions [50, 53]. High temperature stress above 40°C has inhibitory effects on PSII activity by causing denaturation of this protein complex [54]. The assessment of high irradiance on photosynthetic apparatus may play a critical role in understanding potato physiology by providing insights into photosynthetic efficiency and the health of plants [9].

The previous literature indicated that humic acid is one of the important plant biostimulants that contribute to enhancing the growth, yield, and nutrient assimilation in different crops while lessening the dependence on synthetic fertilizers [25, 46, 47, 55]. However, the effect of humic acid on the photosynthetic system and nutrient uptake efficiency of potatoes under high irradiance and in saline soil has never been studied or reported. Despite the fact that our latest work explored microbial‐assisted nitrogen and phosphorus fertilization strategies to enhance the productivity of potato in saline conditions, the present study addresses a fundamentally different research question. In particular, this paper assesses humic acid as an organic biostimulant and elucidates its effect on PSII functionality, energy dissipation mechanisms, and nutrient uptake efficiency in the field with saline sandy loam soil and extended high irradiance. To the best of our knowledge, no prior field experiment has ever quantified chlorophyll fluorescence energy partitioning (Φ_II_, Φ_NPQ_, Φ_NO_, and LEF) and nutrient uptake efficiency in potato in response to humic acid under such environmental conditions. Therefore, this study was conducted to determine the influence of different humic acid application rates on the growth, fluorescence, nutrient uptake, and yield of potato cv. Santana at 65 and 85 days after sowing (DAS) under saline sandy loam soil and high irradiance conditions. The experimental site had 9.1–11.8 sunlight hours per day throughout the period of potato growth (January–April), which is considered a high‐irradiance regime in the subtropical desert climates. This long exposure to intense sunlight each day has more risks of photoinhibition and renders fluorescence‐based assessment very relevant.

## 2. Materials and Methods

### 2.1. Study Site

A field trial was carried out during 2022–23 and 2023–24 at the Horticulture Experimental Area, Department of Horticultural Sciences, Faculty of Agriculture and Environment, The Islamia University of Bahawalpur, Pakistan (29°22′17.4″ N 71°45′53.6″ E). The experimental area has a subtropical climate and is part of the Cholistan desert. This area is well‐known for its extreme heat during the summer. The mean annual rainfall varies between 100 and 200 mm. The rainfall occurs most frequently during the monsoon season (July–September) and the winter/spring season (January–March). The mean annual temperatures fall between 20°C and 40°C. The month‐by‐month average values of air temperature and relative humidity, rainfall, and sunshine were assessed during the crop period (2022–23 and 2023–24). Cholistan’s soils are mainly saline, gypsiferous, and alkaline. The subsurface water is brackish, with over 900 mg L^−1^ salts [56].

Before the experiment, soil samples were collected from the site of the experiment and examined for physicochemical properties using the methodology of Ryan et al. [57]. Soil samples were collected from five different parts of the experimental site before sowing, at 0–15 cm and 16–30 cm depths, using an auger (150 mm high and 20 mm in diameter). The soil obtained from five random cores was mixed. In both layers of soil, the following attributes were measured: pH [58], electrical conductivity (EC) [59], organic matter [60], soil texture [61], available phosphorus [62], available potassium [63], and total nitrogen (N) [64].

### 2.2. Planting Material

Potato cv. Santana, which exhibits taller height with upright stem and foliage structure, dark green leaves, medium to late maturity, high yield, large, long‐oval tuber shape with light yellow skin and cream color flesh, and dry matter around 22.5%, was used as planting material. The certified second‐generation seed material was purchased from a potato market, Okara.

### 2.3. Humic Acid Applications

In the present experiment, three application rates of humic acid, including 1000, 1500, and 2000 kg·ha^−1^ commercially available as “Enrich” manufactured by Syngenta Private Ltd., Pakistan, were compared with an untreated control. The formulation contains 400 g·kg^−1^ humic acid in an 8 kg pack. The aim of selecting a higher dosage of humic acid (2000 kg·ha^−1^) was to evaluate the potential benefits of humic acid applications in saline and alkaline soils where nutrient availability is limited. The humic acid was applied in the form of soil dressing on ridges at 30 and 45 DAS.

### 2.4. Experimental Protocol

Potato crops were grown in two consecutive seasons (2022‐23 and 2023‐24), with planting in January and harvesting in April. During the first growing season, medium‐sized, healthy seed tubers (average weight: 80 ± 5 g) of cv. “Santana” were sown by hand with about 15 cm plant‐plant and 75 cm row‐row spacing at 0.1 m depth of ridges developed by a tractor‐drawn ridge, on January 2, 2022, and tubers were harvested 118 DAS, on April 30, 2022, with the help of a spade. During the second growing season, the sowing was carried out on January 1, 2023, and harvesting on April 26, 2023. Every treatment was reproduced four times, including a control, with a 11.6 m^2^ area for each replication unit. The trial was laid out according to a randomized complete block design (RCBD) with a single or two‐factor factorial setting depending on the studied attributes.

### 2.5. Field and Laboratory Measurements

The attributes related to morphology, fluorescence, and nutrient uptake were recorded from five potato plants selected at random.

#### 2.5.1. Morphological Traits

The growth‐related traits, including plant height (cm), number of stems plant^−1^, number of branches plant^−1^, number of leaves plant^−1^, and leaf area index, were recorded on the 65^th^ and 85^th^ DAS. The measurements taken at 65 and 85 DAS were recorded on the same tagged plants, allowing repeated observations on the same experimental units across both periods. Leaf area was calculated by employing the following formula as previously adopted by Haider et al. [9]. The leaf area index was determined by dividing the estimated leaf area by the ground area covered by the canopy of plants.

(1) Log10 leaf area in cm2=2.060.458×Log10 leaf length cm−.

The yield‐related traits, including number of tubers^−1^ plant, average tuber weight (g), total tuber yield (ton ha^−1^), marketable yield (%), and specific gravity, were recorded after harvesting the potato crop. The marketable tubers comprised those free from defects (cracks, greening, or disease), with a weight greater than 90 g and of uniform shape and size. The specific gravity was determined by the water displacement method.

#### 2.5.2. Chlorophyll Fluorescence Attributes

The traits related to fluorescence, including the quantum yield of PSII (Φ_II_), nonphotochemical quenching (Φ_NPQ_), nonregulatory energy dissipation (Φ_NO_), relative chlorophyll content, and linear electron flow (LEF), were also recorded on the 65^th^ and 85^th^ DAS from 09:00 a.m. to 11:00 a.m. from intact leaves of potato using a MultispeQ Beta instrument and PhotosynQ platform software [65]. Fluorescence measurements at 65 and 85 DAS were taken from the same tagged plants to allow repeated measurements on the same experimental units.

#### 2.5.3. Nutrient Uptake

To determine the N and P uptake, five plants were randomly selected from the experimental area, and their haulm biomass was harvested, weighed, and labelled. In addition, ten tubers were chosen from the harvested area to ensure uniformity and sample size and minimize variability associated with tuber size, weighed, and then sliced into 10 mm wide strips. Initial and final weights were obtained before and after oven‐drying samples of 500 g of tubers and haulm biomass for 72 h at 70°C. The collected samples were further ground using a mortar and pestle for the determination of nutrients (N and P). The phosphorus (P) determination was carried out using the colorimetric method by Murphy and Riley [62] using the UV‐Vis spectrophotometer. The estimation of nitrogen (N) content, on the other hand, was done using the Kjeldhal method as previously described by Bremner [64]. The ground samples were saved in powder form (about 100 g) in plastic bottles for the determination of N (%) and P (%).

## 3. Digestion of the Samples for the Determination of P

One gram of oven‐dried sample was transferred to a 100 mL beaker, and 10 mL of concentrated nitric acid (HNO_3_) was added to it. It was allowed to stand till the initial reaction subsided. It was covered with a watch glass, and low heat was provided until the solid material disappeared. After cooling, to each sample, 5 mL of perchloric acid (HClO_4_) was added, and then again, the sample was heated gently at first and then vigorously until a clear colorless solution resulted. When the volume was reduced to 1 mL, it was cooled and transferred through filter paper to a 50 mL volumetric flask, and the volume was made up to the mark. This filtrate was stored in plastic bottles for further analysis to estimate phosphorus.

### 3.1. Phosphorus (%)

**FIGURE 1:**
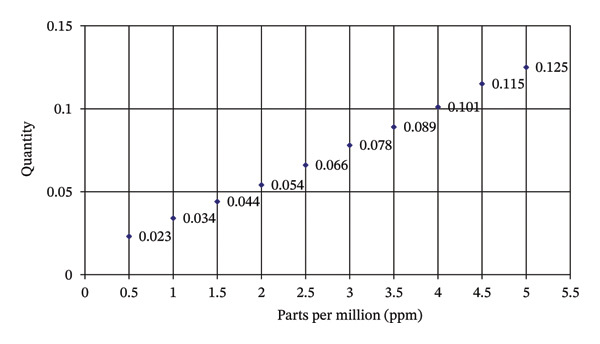
Phosphorus standard curve.

The standard solutions ranging from 0.5 mg·L^−1^ to 5.0 mg·L^−1^ were made at a continuous difference of 0.5 mg·L^−1^. For making a standard solution, the color was developed by adding each of 5% 1:6 H_2_SO_4_, 5% ammonium molybdate, and 0.25% ammonium vanadate. The standard curve (Figure 1) was obtained by using potassium hydrogen phosphate instead of samples. Then, the samples were fed to the spectrophotometer (UV‐2450, Shimadzu, Japan) at a wavelength of 420 nm, and transmittance was noted, which was compared with that of the standard curve to determine the quantity of the element.

The percentage of phosphorus in the samples was calculated by using the following formula:

(2) phosphorus %=ppm on graph × dilution x 100106.

### 3.2. Nitrogen (%)

For the determination of tuber nitrogen, the procedure included digesting the plant material (1 g) with 30 mL concentrated H_2_SO_4_ and 5 g digestion mixture (FeSO_4_: K_2_SO_4_: CuSO_4_ = 1:10:0.5). On cooling, the contents were then transferred to a 250 mL volumetric flask and volume was made up to the mark. Then, 5 mL of aliquot from this prepared material was distilled in micro‐Kjeldhal apparatus using 40% sodium hydroxide 4% boric acid and mixed indicator of methyl red and bromocresol green (BCG). This distillate was titrated against N/10 H_2_SO_4_ till the original color of methyl red was restored. From the quantity of acid used in titration, the percentage of nitrogen was calculated by using the following formula:

(3) nitrogen %=A−B × 1002500.0014 ×  × volume of digested sample used,

where A = quantity of acid used, B = blank reading, and 00014 = constant (which is equal to grams of nitrogen present in 1 mL of N/10 sulfuric acid).

Blank reading was taken for estimating the actual percentage of nitrogen in sample. All the procedures of digestion, distillation, and titration were the same for blank. The N and P nutrient uptake for haulms and tubers was obtained from the product of the dry weight of the tissues and the concentration of the nutrients. The N and P nutrient uptake by the plant was determined by summing the values for both the haulms and tubers.

(4) Nutrient uptake by plant=haulm nutrient uptake+tuber nutrient uptake.

To calculate the efficiency of nutrient uptake by a potato plant, total potato nutrient uptake was divided by nutrient supply using the following formula:

(5) nutrient uptake efficiency=total plant nutrient uptake nutrient supply .

### 3.3. Data Analysis

The data from the two years were averaged using Microsoft Excel 2016. Analysis of variance (ANOVA; homogeneity of variances and normality of residues) assumptions were tested (Shapiro–Wilk and Levene test) with all datasets passing before analysis with suitable transformations. Where necessary, percentage data were arcsine‐transformed, and count data were log‐transformed to meet ANOVA assumptions. A two‐way ANOVA of humic acid rates and years was first performed in order to determine the impact of the year factor. Since the effect of year and its interaction with treatment were nonsignificant (*p* > 0.05), data from both years were pooled, and the means were utilized for final analysis. ANOVA was performed independently on both time intervals (65 and 85 DAS) using Statistix 9 for Windows (Analytical Software, Tallahassee, USA). The mean values were compared using the least significant difference (LSD) test at *p* ≤ 0.05 and p ≤ 0.01. The regression analysis was carried out using R 4.4.0 through the general linear model procedure [66, 67].

## 4. Results

### 4.1. Climate Pattern

**FIGURE 2:**
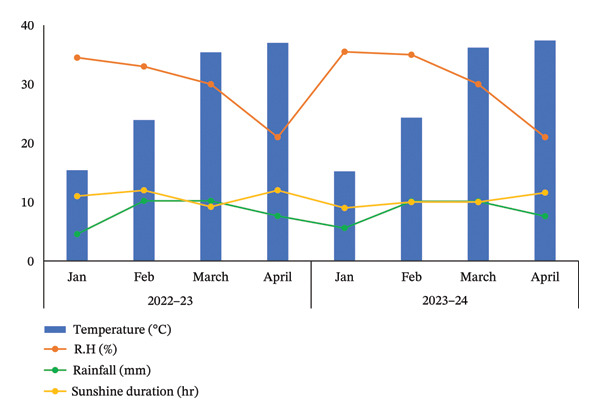
Mean air temperature, relative humidity, rainfall, and sunshine recorded at the experimental site during the period of experiment in 2022‐23 and 2023‐24.

The air temperature increased throughout the experimental period from January to April in both growing seasons (Figure 2). The relative humidity showed a gradual decline from January to March and then a sharp decline in April (Figure 2). The precipitation was initially lower in the first month of the trial and then rapidly increased in the succeeding months (February and March; Figure 2). During both seasons, the sunshine hours slightly changed during the experimental period, ranging from average 9.1 h of sunshine in January to 11.8 h of sunshine in April (Figure 2).

### 4.2. Soil Physicochemical Properties

Table 1 shows the physicochemical properties of the top 15 and 30 cm of the soil before sowing the potato crop. The experimental soil was found to have a medium alkalinity level with pH ranging from 8.0 to 8.1 and EC ranging from 1.70 dS·m^−1^ to 1.85 dS·m^−1^ (Table 1). The sand percentage ranged from 60% to 62% with a texture class of sandy loam (Table 1). The cation exchange capacity (CEC) of the soil varied between 6.5 and 8.1 c mol kg^−1^ (Table 1). This could be due to the buildup of a higher amount of salts (0.71–1.03 g kg^−1^) in the soils of the experimental area (Table 1). The soil organic matter content varied from 0.24% to 0.56% (Table 1). The concentration of N varied between 0.048% and 0.059% (Table 1). The amount of available P and K in the soil ranged between 0.39–0.67 mg kg^−1^ and 52–84 mg kg^−1^, respectively (Table 1). The selected physicochemical properties of irrigated water are presented in Table 2. The irrigated water also had an intermediate sodicity and alkalinity level (Table 2).

**TABLE 1:** The physicochemical examination of the soil at the experimental site during 2022–23 and 2023–24.

Particular	Unit	Values at two different soil depths	
		0–15 cm	16–30 cm
Soil texture	—	Sandy loam	Sandy loam
Sand	%	60	62
Silt	%	24	27
Clay	%	16	11
Saturation	%	34	34
EC	dS·m^−1^	1.85	1.70
pH	—	8.0	8.1
Total dissolved salts	g·kg^−1^	0.93	0.72
Organic matter	%	0.56	0.24
CEC	c mol·kg^−1^	8.1	6.5
Total N	%	0.059	0.048
Available P	mg kg^−1^	0.67	0.39
Available K	mg kg^−1^	84	52

**TABLE 2:** Physicochemical analysis of irrigated water used in the experiment during 2022‐23 and 2023‐24.

Particular	Unit	Value
pH	—	7.5
EC	dS m^−1^	1.18
Total dissolved salts	g L^−1^	1.15
Ca^+2^ + Mg^+2^	Meq L^−1^	7.30
Na^+^	Meq L^−1^	4.85
CO_3_^−2^	Meq L^−1^	—
HCO_3_^−1^	Meq L^−1^	2.57
Cl^−1^	Meq L^−1^	1.10
Sodium adsorption ratio	—	2.47
Residual sodium carbonate	Meq L^−1^	0.21

### 4.3. Morphological Traits in Potato cv. Santana

In the present study, the individual effects of humic acid application rates and periods were found to be highly significant (*p* ≤ 0.01) for plant height, number of stems plant^−1^, number of branches plant^−1^, number of leaves plant^−1^, and leaf area index (Table S1). The interactive effect of humic acid application rates and periods was significant (*p* ≤ 0.05) for the number of stems and branches plant^−1^ and highly significant (*p* ≤ 0.01) for plant height, number of leaves plant^−1^, and leaf area index (Table S1). The above morphological traits showed an increasing trend with an increase in humic acid application rates (Figures 3(a), 3(b), 3(c), 3(d), and 3(e)). The highest plant height was attained at 85 DAS with the application of the highest rate of humic acid (2000 kg·ha^−1^), about threefold higher compared to the untreated control at 65 DAS (Figure 3(a)). Similarly, the highest numbers of stems, branches, and leaves plant^−1^ were obtained at 85 DAS with the application of the highest rate of humic acid (2000 kg ha^−1^), about 3.6, 1.7, and 2.1 times higher compared to the untreated control at 65 DAS, respectively (Figures 3(b), 3(c), and 3(d)). The greatest leaf area index was also noted at 85 DAS with the application of the highest rate of humic acid, about 3.4 times higher compared to the untreated control at 65 DAS (Figure 3(e)). Furthermore, the application of humic acid was found to have a highly significant (*p* ≤ 0.01) effect on tuber number plant^−1^, tuber weight, total and marketable tuber yield, and specific gravity (Table S2). These traits showed improvement with an increase in the rate of humic acid (Figures 3(f), 3(g), 3(h), 3(i), and 3(j)). The highest numbers of tubers plant^−1^, average tuber weight, total tuber yield, marketable yield, and specific gravity were achieved with the application of the highest rates of humic acid (2000 kg·ha^−1^), about 2.2, 1.3, 1.6, 1.5, and 1.1 times higher compared to the untreated control, respectively (Figures 3(f), 3(g), 3(h), 3(i), and 3(j)).

FIGURE 3Plant height (a), number of stems plant^−1^ (b), number of branches plant^−1^ (c), number of leaves plant^−1^ (d), leaf area index (e), number of tubers plant^−1^ (f), average tuber weight (g), total tuber yield (h), yield (i), and specific gravity (j) of potato cv. Santana receiving 0, 1000, 1500, and 2000 kg·ha^−1^ humic acid at 30 and 45 days after sowing. Each time point in this trial was analyzed independently. The vertical error bars represent the mean values (± standard error) averaged over two years and four replications. Since no significant year effect was observed, the data were pooled across years. Lettering is used to express the differences within the means of treatments conducted by least significant difference (LSD) test at the *p* ≤ 0.05 after one‐way analysis of variance. Sample size (*n*) = 4  ×  4 (HA  ×  rep) = 16. HA = humic acid.

**Figure:**
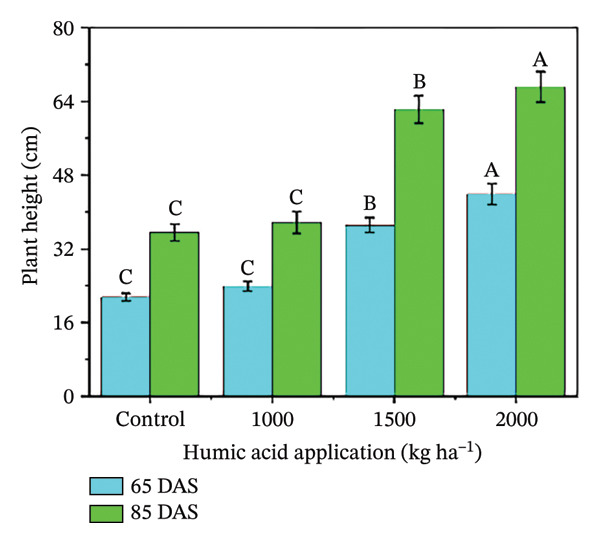
(a)

**Figure:**
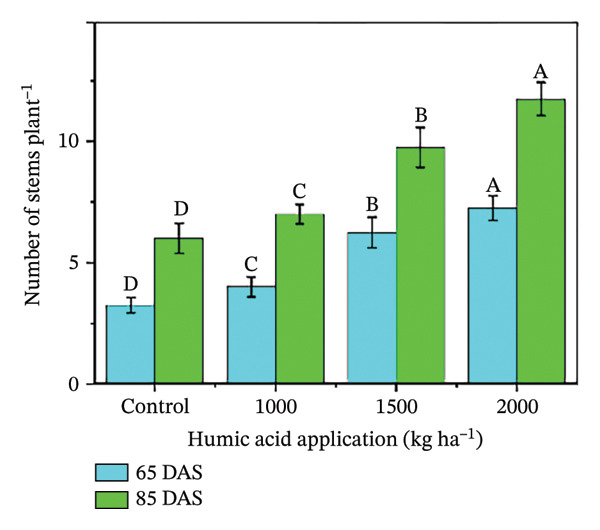
(b)

**Figure:**
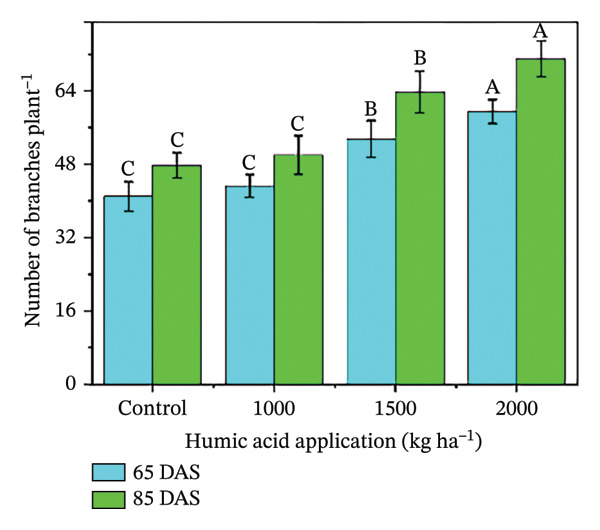
(c)

**Figure:**
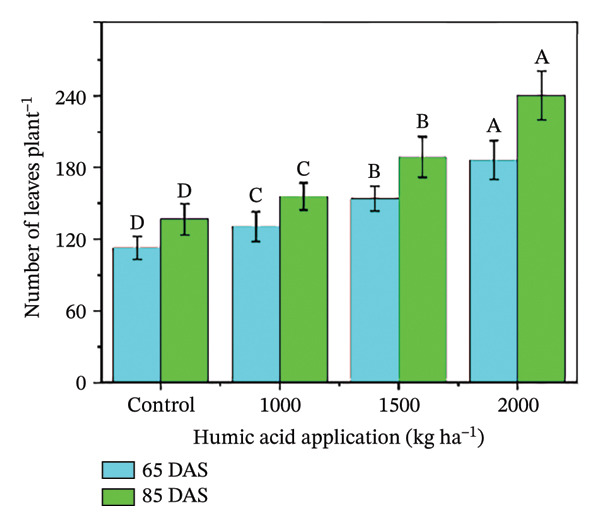
(d)

**Figure:**
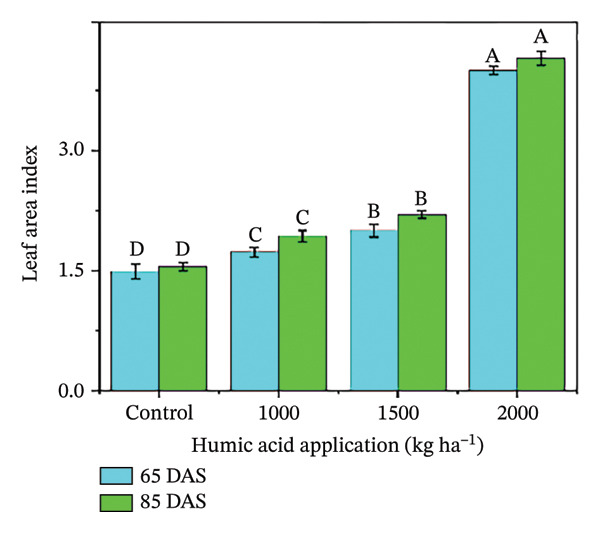
(e)

**Figure:**
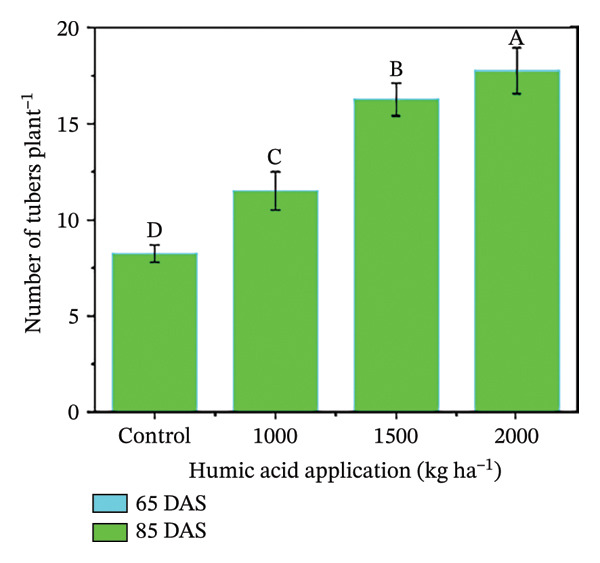
(f)

**Figure:**
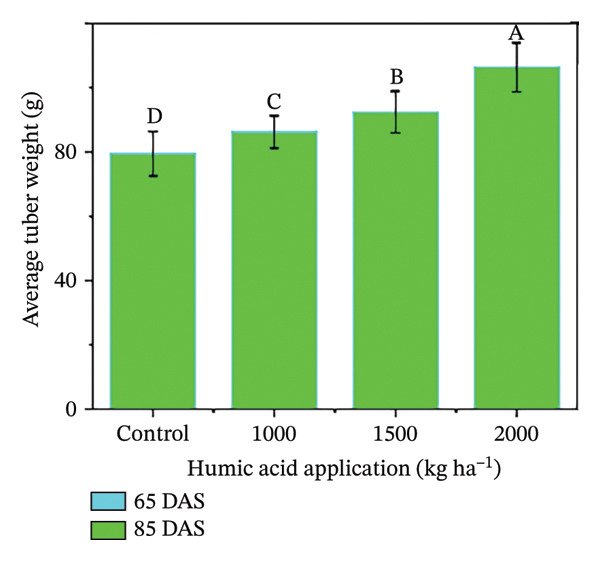
(g)

**Figure:**
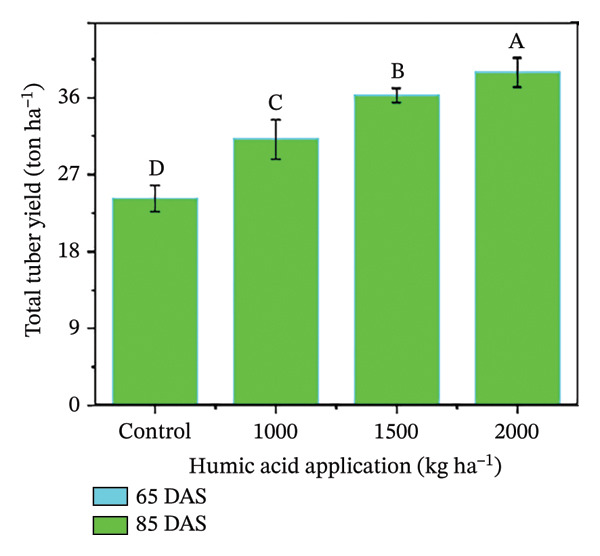
(h)

**Figure:**
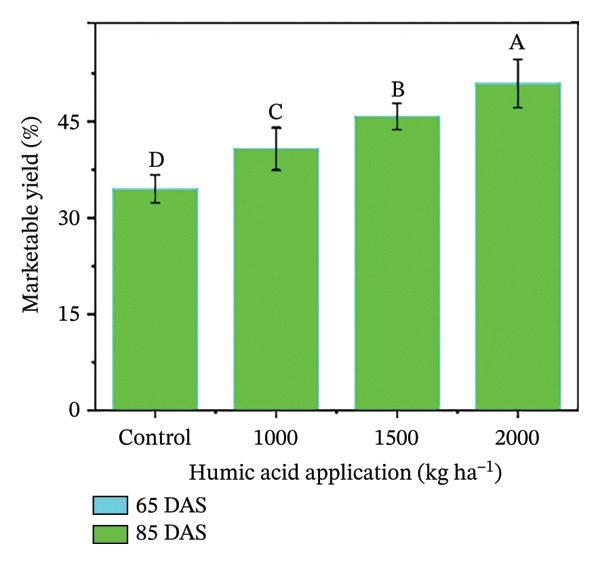
(i)

**Figure:**
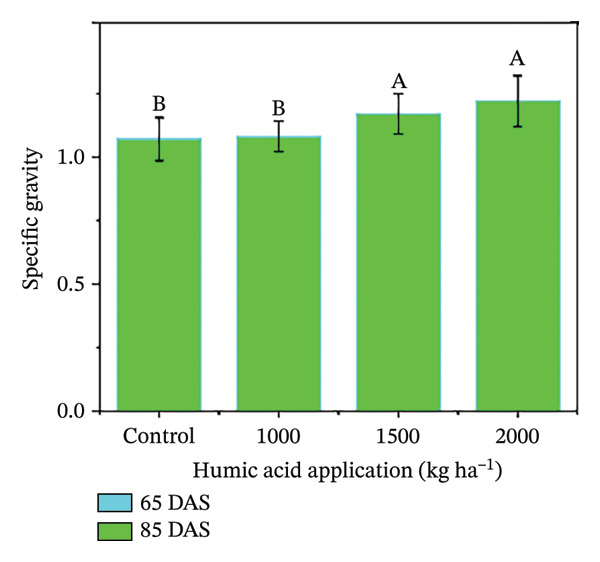
(j)

### 4.4. Fluorescent Traits in Potato cv. Santana

The fluorescent traits in potatoes are crucial to determine for understanding their physiological responses and improving nutrient management. The highly significant (*p* ≤ 0.01) variations were observed for quantum yield of PSII (Φ_II_), relative chlorophyll content, nonphotochemical quenching (Φ_NPQ_), and nonregulatory energy dissipation (Φ_NO_) under the main and interactive effects of humic acid application rates and periods (Table S3). LEF was found to be highly significant (*p* ≤ 0.01) for the main effects and significant (*p* ≤ 0.05) for the interactive effect (Table S3). The values of Φ_II_, relative chlorophyll content, and LEF increased with an increment in humic acid (Figures 4(a), 4(b), and 4(c)). The values of Φ_NPQ_ and Φ_NO_, on the other hand, decreased (Figures 4(d) and 4(e)). Regarding the period, Φ_II_, relative chlorophyll content, and LEF were found to have higher values at 85 DAS than 65 DAS and vice versa for Φ_NO_ (Figures 4(a) and 4(e)). While Φ_NPQ_ showed a different response, it was higher at 85 DAS with the application of 0–2000 kg·ha^−1^ than at 65 DAS, but was found lower at 85 DAS with an application of 1500 and 2000 kg·ha^−1^ humic acid than at 65 DAS (Figure 4(d)). The highest value for Φ_II_ was recorded at 85 DAS with the application of the highest rate of humic acid (2000 kg·ha^−1^), about threefold greater compared to the untreated control at 65 DAS (Figure 4(a)). Despite this, the relative chlorophyll content and LEF were also found to be highest with the application of the highest rate of humic acid (2000 kg·ha^−1^), at 85 DAS, approximately 1.8 and 2.2 times higher in comparison with the untreated control at 65 DAS, correspondingly (Figures 4(b) and 4(c)). Alternatively, the values of Φ_NPQ_ and Φ_NO_ were found to be lowest with the application of the highest rate of humic acid (2000 kg·ha^−1^), at 85 DAS, approximately 4.4 and 2.6 times smaller in comparison with the untreated control at 65 DAS, respectively (Figures 4(d) and 4(e)).

FIGURE 4The fluorescence‐related traits including quantum yield of photosystem II (ф_II_) (a),relative chlorophyll content (b), linear electron flow (c), nonphotochemical quenching (ф_NPQ_) (d), and nonregulatory energy dissipation (ф_NO_) (e) recorded in potato plants cv. Santana receiving 0, 1000, 1500, and 2000 kg·ha^−1^ humic acid at 30 and 45 days after sowing. Each time point in this trial was analyzed independently. The vertical error bars represent the mean values (± standard error) averaged over two years and four replications. Since no significant year effect was observed, the data were pooled across years. Lettering is used to express the differences within the means of treatments conducted by least significant difference (LSD) test at the *p* ≤ 0.05 after one‐way analysis of variance. Sample size (*n*) = 4  ×  4 (HA  ×  rep) = 16. HA = humic acid.

**Figure:**
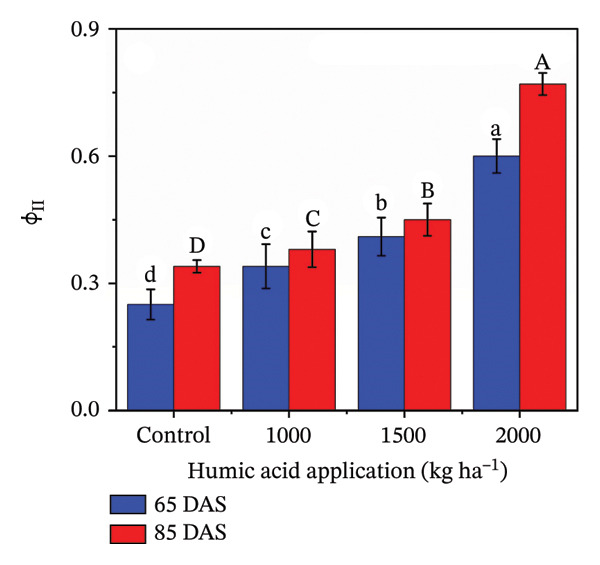
(a)

**Figure:**
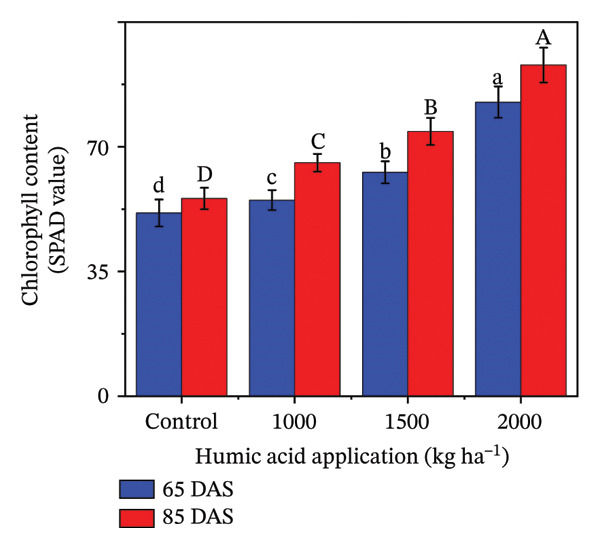
(b)

**Figure:**
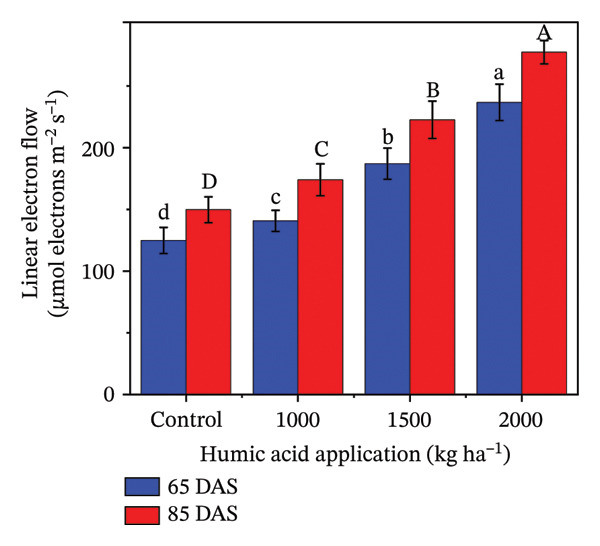
(c)

**Figure:**
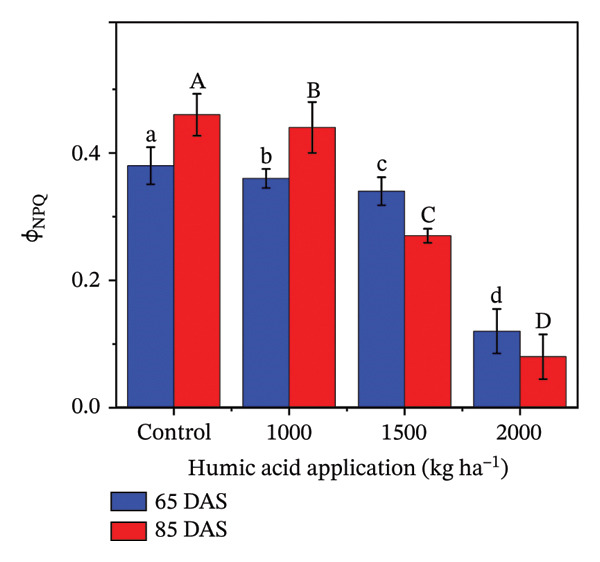
(d)

**Figure:**
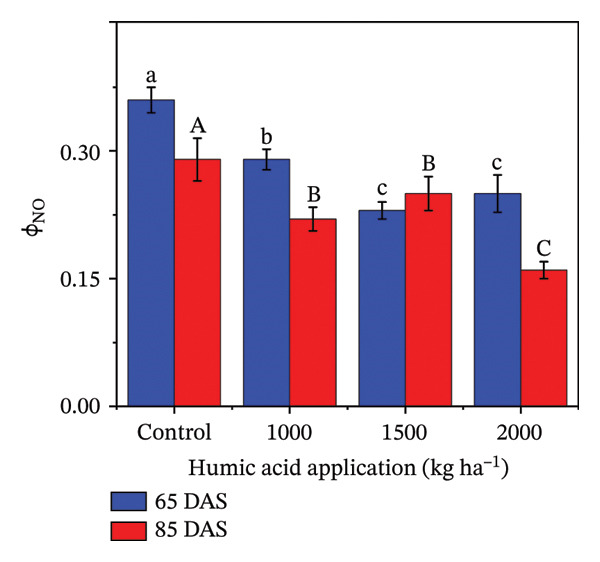
(e)

### 4.5. Nutrient Uptake in Potato cv. Santana

The determination of nutrient uptake in the potato crop is essential for optimizing fertilization strategies and enhancing crop yield and quality. The individual and combined effects of both humic acid application rates and periods were found to have a highly significant effect (*p* ≤ 0.01) on total plant N uptake, N uptake efficiency (NUE), total plant P uptake, and P uptake efficiency (PUE) (Table S4). The inclining trend was noticed for nutrient uptake‐related traits with the increase in the application rate of humic acid (Figures 5(a), 5(b), 5(c), and 5(d)). Total plant N and P uptake boosted by 24.7% and 73.2% in the plot supplemented with 2000 kg·ha^−1^ humic acid compared to the control, respectively (Figures 5(a) and 5(c)). Additionally, the application of 2000 kg·ha^−1^ also enhanced the NUE and PUE by approximately 1.5 and 2.8 times compared to the untreated control, respectively (Figures 5(b) and 5(d)).

FIGURE 5Nitrogen uptake (a), efficiency of nitrogen uptake (NUE) (b), phosphorus uptake (c), and efficiency of phosphorus uptake (PUE) (d) assessed in potato cv. Santana receiving 0, 1000, 1500, and 2000 kg·ha^−1^ humic acid at 30 and 45 days after sowing. The vertical error bars represent the mean values (± standard error) averaged over two years and four replications. Since no significant year effect was observed, the data were pooled across years. Lettering is used to express the differences within the means of treatments conducted by least significant difference (LSD) test at the *p* ≤ 0.05 after the analysis of variance. Sample size (*n*) = 4  ×  4 (HA  ×  rep) = 16. HA = humic acid.

**Figure:**
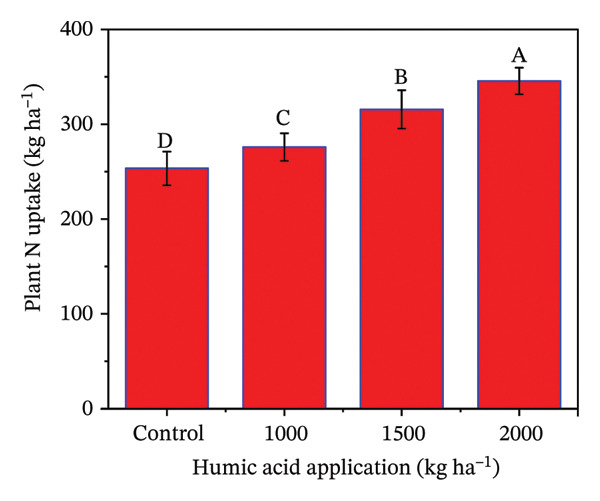
(a)

**Figure:**
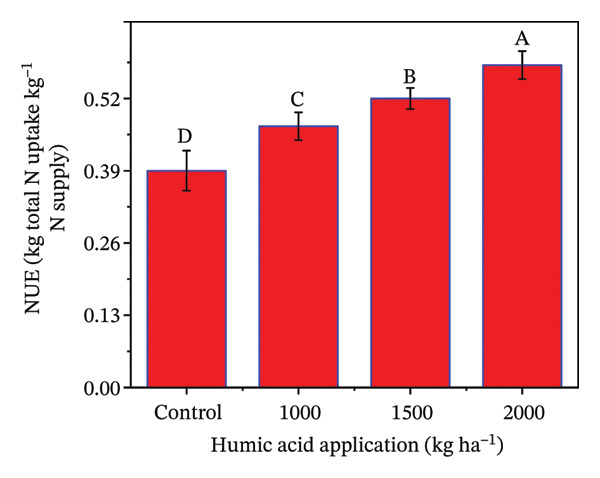
(b)

**Figure:**
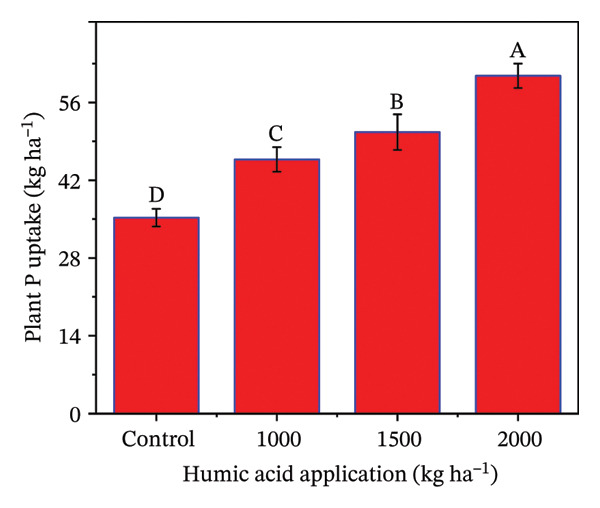
(c)

**Figure:**
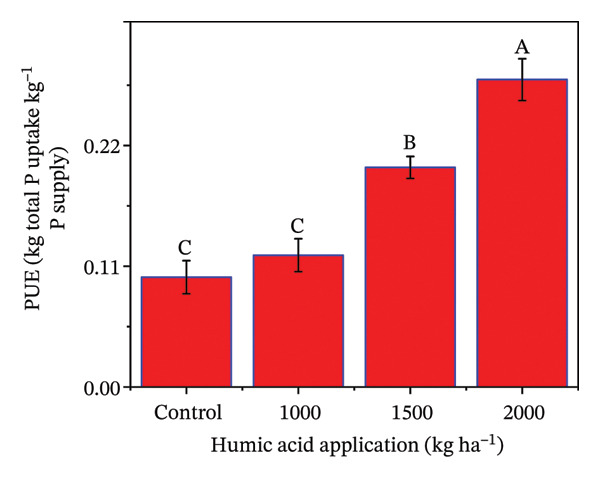
(d)

## 5. Discussion

**FIGURE 6:**
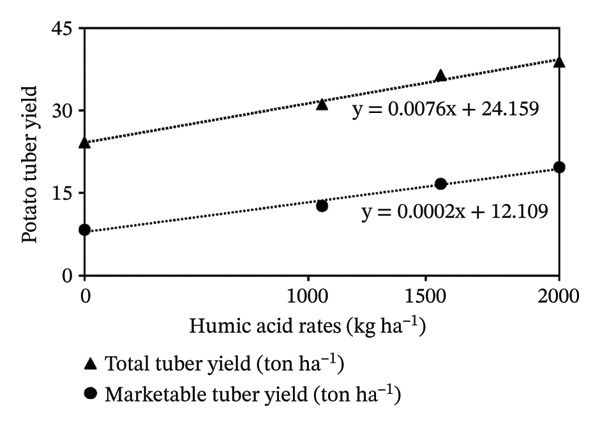
Regression analysis of total tuber yield and marketable yield of potato cv. Santana with the application rates of humic acid.

The growth parameters have a direct impact on plant yield. In this study, the individual and interactive effect of humic acid with the period was found to be substantial on the growth of potato cv. Santana. The results showed that with an increase in the application rate of humic acid, all these traits showed an increasing trend; however, their highest values were attained with the application of 2000 kg·ha^−1^ (Figures 2, 3, and 4). We observed that the soil dressing of humic acid (1000–2000 kg·ha^−1^) resulted in greater plant and tuber growth of the potato cv. Santana and hence increased yields, similar to the results of the studies conducted by Ekin [25] and Suh et al. [49]. Since the period from mid‐February to mid‐March during both growing seasons (2022‐23 and 2023‐24) was relatively wet (Figure 1), likely much of the in‐season application of N and P would have been lost to leaching, particularly on the sandy loam soils of this study. A more balanced approach to fertilization, such as the application of higher doses of humic acid (1500–2000 kg·ha−1) when the plants most require N and P at times, and when rainfall is less, and hence, reduced leaching due to humic acid application in the soil. In the present study, total and marketable yields showed a positive linear regression with an increased application rate of humic acid, reaching its maximum at the highest rate of 2000 kg·ha^−1^ (Figure 6). This linear increase suggests that humic acid effectively enhances nutrient availability, which may lead to improved root development, nutrient uptake, and stress tolerance. Higher dosages of humic acid likely facilitated better retention of moisture and nutrients in the saline and alkaline soils, contributing to the optimal growth conditions for potatoes. Additionally, the positive correlation between humic acid application and yield aligns with other studies [68–72], showing that humic substances promote crop growth by improving photosynthetic efficiency and mitigating the detrimental effects of abiotic stress. However, marketable yield showed a stronger dependence on humic acid application rates than total tuber yield (Figure 6). The specific gravity of the potato tuber is a key quality trait that determines the product pricing [73, 74]. In the present investigation, the increase in specific gravity with an increasing rate of humic acid application suggests that it improved nutrient uptake, hence leading to a high dry matter content as compared with control plants. Our findings corroborate the previous work of Selim et al. [75], who noted a significant increase in the specific gravity of potato tubers with the addition of humic acid.

Chlorophyll content represents the health of plants and is also used to study the efficiency of the photosynthetic organelles [9]. High temperature during the cropping season (Figure 1), particularly at the tuber initiation stage, which initiates within 45 DAS [76], can impair PSII function and reduce chlorophyll content, leading to lower fluorescence yield and reduced photosynthetic efficiency [77–80]. The salinity exacerbates nutrient imbalances, causing stress that inhibits root absorption of essential nutrients like nitrogen and phosphorus. However, humic acid played a critical role in mitigating these adverse effects by enhancing the plants’ tolerance to heat stress through improved chlorophyll content and Φ_II_ (Figure 3(d)). The improvement in the relative chlorophyll content of potato plants may be attributed to improved soil saturation, nutrient availability, and root oxygenation [9, 81, 82]. Φ_II_ is essentially the percentage of incoming light (excited electrons) that goes into PSII, where most light energy is converted into food. In the current investigation, Φ_II_ was 2.4 times greater in the plants that received 2000 kg·ha^−1^ humic acid than those that received no humic acid (control) (Figure 3(a)). A linear relationship has been documented between Φ_II_ and relative chlorophyll content in numerous reports [83–85]. Φ_NPQ_ is the percentage of light that goes towards nonphotochemical quenching. The plant utilizes this energy to combat any stress or damage. A significant variation between the humic acid treatments for Φ_NPQ_ (Table S3) is in good agreement with the past investigations [86, 87]. Φ_NO_ is the percentage of light that is lost via nonregulated processes. Φ_NO_ is the combination of some unregulated processes whose by‐products can inhibit photosynthesis or be harmful to the plant. In this research, Φ_NO_ was noticed to be 1.8 times lower in the plants receiving 2000 kg·ha^−1^ humic acid compared to the untreated control plants. Electron transport regulation also maintains a balance between the availability of light and metabolic requirements. LEF is the total flow of electrons from the antenna complexes, where light is captured, into PSII. LEF plays a critical role in plant growth by establishing a pH gradient across the thylakoid membrane (ΔpH) [88]. This gradient promotes adenosine triphosphate (ATP) synthesis and activates Φ_NPQ_ for protection during stress [89]. In the current study, the plants received 2000 kg·ha^−1^ humic acid which was noted to have a comparatively greater LEF, two‐fold of that was recorded in untreated control plants (Figure 3(e)).

The application of humic acid played a pivotal role in improving soil structure, which enhanced water retention and root penetration [90, 91], allowing for more efficient nutrient absorption. Additionally, humic acid acts as a natural chelator, binding to essential nutrients such as N, P, and K ions and maintaining their immediate availability to the plant [25]. This increased nutrient availability supported better root growth and metabolic activity, particularly in saline and alkaline soils, which had nutrient leaching and poor nutrient availability problems. Moreover, humic acid stimulates microbial activity in the rhizosphere, promoting the mineralization of organic matter and facilitating the uptake of nutrients. Resultantly, N uptake (1.4 times), P uptake (1.7 times), NUE (1.5 times), and PUE (2.8 times) were considerably increased in the plants receiving 2000 kg·ha^−1^ compared to the untreated control (Figures 4(a), 4(b), 4(c), and 4(d)). The findings are comparable to those of Mesut et al. [92], who reported that humic acid application significantly increases the uptake of N, P, K, Ca, and Mg in pepper seedlings. Similarly, in another study, Heba et al. [93] reported that humic acid had a positive effect on nutrient uptake in barley. Our results are also supported by Esringü et al. [47], who found significant variations between humic acid treatments for nutrient uptake in Hungarian vetch. The improved nutrient uptake might be attributed to increase in CEC, improved soil aggregation, and enhanced water retention by humic acid application, which together reduced nutrient loss and promoted greater mobility of ammonium, nitrate, and phosphate ions [43, 89]. Moreover, humic molecules also act as natural chelators, forming complexes with macro‐ and micronutrients that prevent their precipitation and maintain them in plant‐available forms [25, 68]. The latest findings further demonstrate that humic acid stimulates rhizosphere microbial activity, accelerates mineralization of organic matter, and activates root plasma‐membrane H^+^‐ATPase, thereby increasing nutrient absorption capacity even under abiotic stress [67, 90, 92]. Although the present study indicates clear benefits of humic acid in improving growth, fluorescence, and nutrient uptake of potato under saline sandy loam soil, further studies should concentrate on direct measurements of soil nutrient availability and nutrient fractions to confirm the pathways through which humic acid enhances nutrient uptake under saline conditions.

## 6. Conclusion

From the above results, it can be concluded that humic acid remarkably improved morphology, fluorescence, and nutrient uptake in potato cv. Santana and hence can be used as a protectant to enhance growth and development under high temperatures and in saline soil. However, an additional study is required to test one more interval to stabilize the rate of humic acid. Furthermore, scaling up this research to other varieties of potato could validate its broader applicability and provide varietal‐specific recommendations.

## Author Contributions

Muhammad Wasim Haider: conceptualization, formal analysis, investigation, methodology, and writing–original draft. Syed Mohsin Abbas: conceptualization, project administration, visualization, and resources. Tanveer Hussain: conceptualization, resources, and visualization. Muhammad Tahir Akram: writing–review and editing, methodology, formal analysis, and data curation. Muhammad Asad Saleem: writing–review and editing, validation, and methodology. Muhammad Waseem: methodology, formal analysis, writing–original draft, writing–review and editing, investigation, visualization, and validation. Alina‐Stefania Stanciu: methodology, formal analysis, and writing–review and editing. Muhammad Nafees: writing–review and editing, visualization, methodology, and investigation. Crossby Osei Tutu: formal analysis, writing–review and editing, software, and visualization.

## Funding

The authors are grateful to the Department of Horticultural Sciences at The Islamia University of Bahawalpur for facilitating this field experiment at the Experimental Area. The Higher Education Commission (HEC) of Pakistan provided funds for research material under the Project No. SRGP/NAHE/HEC/2020/286, entitled “Improving Potato Production Through Varietal and Fertilizer Management Under Cholistan Region.”

## Ethics Statement

This study does not include human or animal subjects. The seed material was purchased from a potato market, Okara. The field and laboratory studies were carried out with all applicable institutional, national, and international guidelines and legislation. The sampling permissions or licenses were obtained by the landowner.

## Conflicts of Interest

The authors declare no conflicts of interest.

## Supporting Information

Table S1. Analysis of variance for plant height, number of stems plant^−1^, number of branches plant^−1^, number of leaves plant^−1^, and leaf area index in potato plants at 65^th^ and 85^th^ DAS after application of 0, 1000, 1500, and 2000 kg·ha^−1^ humic acid.

Table S2. Analysis of variance for number of tubers plant^−1^, average tuber weight, total tuber yield, marketable yield, and specific gravity in potato after application of 0, 1000, 1500, and 2000 kg·ha^−1^ humic acid.

Table S3. Analysis of variance for quantum yield of PSII (**ϕ**_**II**_), relative chlorophyll content, LEF, nonphotochemical quenching (**ϕ**_**NPQ**_), and nonregulatory energy dissipation (**ϕ**_**NO**_) in potato plants at 65^th^ and 85^th^ DAS after application of 0, 1000, 1500, and 2000 kg·ha^−1^ humic acid.

Table S4. Analysis of variance for plant N uptake, NUE, plant P uptake, and PUE in potato after application of 0, 1000, 1500, and 2000 kg·ha^−1^ humic acid.

## Supporting information


**Supporting Information** Additional supporting information can be found online in the Supporting Information section.

## Data Availability

All the data related to this work can be sourced from the corresponding authors.
