# Supplementary material for: Effect of Humic Acid on Morphology, Fluorescence, and Nutrient Uptake of Spring‐Sown Potato Crop Under Saline Sandy Loam Soil
Source: Scientifica (Cairo). 2026 Mar 1;2026:6184394. doi: 10.1155/sci5/6184394 (PMC12950902; doi:10.1155/sci5/6184394)
Supplement: Supplementary file 1 — Supporting Information Additional supporting information can be found online in the Supporting Information section. [file SCI5-2026-6184394-s001.docx]

**Table S1.** Analysis of variance for plant height, number of stems plant^–1^, number of branches plant^–1^, number of leaves plant^–1^, and leaf area index in potato plants at 65^th^ and 85^th^ day of sowing after application of 0, 1000, 1500, and 2000 kg ha^–1^ humic acid.

| **Source of variance** | **Plant**  **height** | **Number of stems plant^–1^** | **Number of branches plant^–1^** | **Number of leaves plant^–1^** | **Leaf area index** |
| --- | --- | --- | --- | --- | --- |
|  | **Percentage of total variance** | | | | |
| Humic acid (HA) | 57.54** | 54.48** | 74.30** | 74.82** | 89.65** |
| Interval (I) | 38.32** | 40.27** | 21.58** | 20.04** | 2.25** |
| HA × I | 2.81** | 3.68* | 2.78* | 2.47** | 2.38** |
| Error | 1.05 | 1.53 | 1.12 | 1.32 | 1.47 |

* Significant at *P ≤ 0.05*.

** Significant at *P ≤ 0.01*

**Table S2.** Analysis of variance for number of tubers plant^–1^, average tuber weight, total tuber yield, marketable yield, and specific gravity in potato after application of 0, 1000, 1500, and 2000 kg ha^–1^ humic acid.

| **Source of variance** | **Number of tubers plant^–1^** | **Average tuber weight** | **Total tuber yield** | **Marketable yield** | **Specific gravity** |
| --- | --- | --- | --- | --- | --- |
|  | **Percentage of total variance** | | | | |
| Humic acid (HA) | 93.98** | 96.89** | 98.66** | 96.78** | 82.71** |
| Error | 5.66 | 2.58 | 1.14 | 2.43 | 13.86 |

** Significant at *P ≤ 0.01*

**Table S3.** Analysis of variance for quantum yield of photosystem II (**ϕ_II_**), relative chlorophyll content, linear electron flow, non-photochemical quenching (**ϕ_NPQ_**), and non-regulatory energy dissipation (**ϕ_NO_**) in potato plants at 65^th^ and 85^th^ day of sowing after application of 0, 1000, 1500, and 2000 kg ha^–1^ humic acid.

| **Source of variance** | **ϕ_II_** | **Relative chlorophyll**  **content** | **Linear electron flow** | **Φ_NPQ_** | **Φ_NO_** |
| --- | --- | --- | --- | --- | --- |
|  | **Percentage of total variance** | | | | |
| Humic acid (HA) | 87.53** | 86.09** | 85.89** | 93.15** | 20.95** |
| Interval (I) | 6.97** | 10.83** | 11.32** | 0.45** | 45.37** |
| HA × I | 3.61** | 1.74** | 0.85* | 6.06** | 26.91** |
| Error | 2.54 | 1.13 | 0.32 | 0.18 | 5.87 |

* Significant at *P ≤ 0.05*.

** Significant at *P ≤ 0.01*

**Table S4.** Analysis of variance for plant N uptake, N uptake efficiency (NUE), plant P uptake, and P uptake efficiency (PUE) in potato after application of 0, 1000, 1500, and 2000 kg ha^–1^ humic acid.

| **Source of variance** | **Plant N uptake** | **NUE** | **Plant P uptake** | **PUE** |  |
| --- | --- | --- | --- | --- | --- |
|  | **Percentage of total variance** | | | | |
| Humic acid (HA) | 97.17** | 92.95** | 96.88** | 88.92** |  |
| Error | 1.59 | 5.28 | 2.64 | 9.16 |  |

** Significant at *P ≤ 0.01*
